# Trends in Maternal Outcomes During the COVID-19 Pandemic in Alabama From 2016 to 2021

**DOI:** 10.1001/jamanetworkopen.2022.2681

**Published:** 2022-04-13

**Authors:** Vivek V. Shukla, AKM Fazlur Rahman, Xuejun Shen, Allison Black, Arie Nakhmani, Namasivayam Ambalavanan, Waldemar A. Carlo

**Affiliations:** 1 Department of Pediatrics, University of Alabama at Birmingham, Birmingham; 2 Department of Biostatistics, University of Alabama at Birmingham, Birmingham; 3 Alabama Department of Public Health, Montgomery; 4 Department of Electrical and Computer Engineering, University of Alabama at Birmingham, Birmingham

## Abstract

This cohort study assesses whether the COVID-19 pandemic is associated with an increase in the risk of maternal morbidity and mortality in Alabama from 2016 to 2021.

## Introduction

The COVID-19 pandemic has been associated with worse health outcomes in patients infected with COVID-19 and patients who were not infected,^1,2,3^ including worse pregnancy-related outcomes.^4^ This population-based study with individual participant data covers the period from before the pandemic (2016-2019) to during the pandemic (from March 2020 to September 2021) and is the first, to our knowledge, to compare the risk of maternal mortality and morbidities in the initial and Delta pandemic periods with the baseline period.^4^ This study assesses whether the COVID-19 pandemic is associated with an increase in the risk of maternal mortality.

## Methods

This population-based cohort study was conducted using the Alabama Department of Public Health Center for Health Statistics database, a population-based state vital registry. The time frame was defined in terms of calendar years, months, and numbered weeks. Calendar weeks were defined beginning from week 1 as the first Sunday of the year. We compared pregnancies from the following 3 periods: baseline (2016-2019, January-December, weeks 1-52, and 2020, January-February, weeks 1-8), initial pandemic (2020, March-December, weeks 9-52, and 2021, January-June, weeks 1-26), and Delta pandemic (2021, July-September, weeks 27-39).

All Alabama State resident deliveries in Alabama with stillbirths at 20 weeks or more gestation or live births at 22 weeks or more gestation were included. The database definitions were used to define outcomes and variable categories (eMethods in the Supplement).^5^ The study was approved by the institutional review board at the University of Alabama at Birmingham with a waiver of informed consent because the study was based on a deidentified public health database. This study followed the Strengthening the Reporting of Observational Studies in Epidemiology (STROBE) reporting guideline for reporting observational studies.

Descriptive analyses were used to describe characteristics and outcomes. The χ^2^, Mann-Whitney U, or *t* tests was used, as appropriate, to compare characteristics between the baseline and pandemic periods. Multivariable logistic regression was performed for maternal mortality, adjusting for maternal age, race and ethnicity (as reported by parents or health care professionals, identified from birth or death certificates, and classified per the National Center for Health Statistics), and educational level. A 2-sided *P* value <.05 was used to indicate statistical significance. All analyses were performed in SAS version 9.4 (SAS Institute).

## Results

A total of 325 036 pregnancies were included; 236 481 were from the baseline period, 74 076 from the initial pandemic period, and 14 479 from the Delta pandemic period (Table). The maternal mortality rate showed distinct peaks in May 2020 (rate of 194.9 per 100 000 births), November to December 2020 (rates of 213.1 and 228.9 per 100 000 births), and during the Delta pandemic period of July to September 2021, (rates of 199.6, 194.6, and 231.6 per 100 000 births). The mean maternal mortality rates increased in the initial pandemic period and further increased in the Delta pandemic period in unadjusted analysis (65.1 to 109.3 and 207.2 per 100 000 births; both *P* < .001) (Figure) and adjusted analysis (initial pandemic vs baseline period, adjusted odds ratio, 1.6; 95% CI, 1.2-2.1; *P* = .001; Delta pandemic vs baseline period, adjusted odds ratio, 3.7; 95% CI, 2.5-5.3; *P* < .001; and Delta pandemic vs initial pandemic period, adjusted odds ratio, 2.3; 95% CI, 1.5-3.5; *P* < .001).

**Table.** Sociodemographic Characteristics and Maternal Outcomes

Variable	No. (%)			*P* value
	Baseline period^a^ (n = 236 481)	Pandemic period^b^		
		Initial (n = 74 076)	Delta (n = 14 479)	
Maternal age, median (IQR), y	27 (23-31)	28 (23-32)	28 (23-32)	<.001
Maternal educational level				
<High school	34 968 (14.8)	9997 (13.5)	1884 (13.0)	<.001
High school graduate	74 690 (31.6)	24 453 (33.0)	4813 (33.2)	
Some college/associate degree	70 450 (29.8)	20 885 (28.2)	4085 (28.2)	
Bachelor degree	35 948 (15.2)	11 554 (15.6)	2331 (16.1)	
Master/PhD/professional degree	19 907 (8.4)	7107 (9.6)	1345 (9.3)	
Unknown	518 (0.2)	80 (0.2)	21 (0.15)	
Maternal race and ethnicity				
Black	72 927 (30.8)	22 173 (29.9)	4143 (28.6)	<.001
Hispanic	11 777 (5.0)	3886 (5.2)	794 (5.5)	
White	141 436 (59.8)	44 074 (58.5)	8723 (60.2)	
Other^c^	10 341 (4.4)	3943 (5.3)	819 (5.7)	
Adequate prenatal care	140 653 (59.5)	43 792 (58.1)	8709 (60.2)	<.001
Gestational diabetes	12 322 (5.2)	4572 (6.3)	977 (6.8)	<.001
Pregnancy-induced hypertension	21 373 (9.0)	8603 (11.6)	1711 (11.8)	<.001
Maternal blood transfusion	735 (0.3)	363 (0.5)	67 (0.5)	<.001
Uterine rupture	80 (0.03)	23 (0.03)	3 (0.02)	.67
Unplanned hysterectomy	236 (0.1)	54 (0.07)	9 (0.06)	.05
Maternal ICU admission	310 (0.1)	86 (0.1)	23 (0.2)	.36
Maternal deaths, No. per 100 000 births	154 (65.1)	81 (109.3)	30 (207.2)	<.001
Cesarean delivery (live births)	81 380 (34.7)	25 701 (35.0)	5094 (35.5)	.09

Abbreviation: ICU, intensive care unit.

^a^ The baseline period is defined as the years before the pandemic, from 2016 to 2019 and the first 8 weeks of 2020.

^b^ The pandemic period is defined as the initial pandemic (March 2020 to June 2021) and the Delta pandemic (July to September 2021).

^c^ Race and ethnicity including American Indian, Asian Indian, Chinese, Filipino, Guamanian or Chamorro, Samoan, Japanese, Korean, Native Alaskan, Native Hawaiian, Native Pacific Islander, Vietnamese, other Pacific Islander, and other Asian were classified as other.

**Figure.**
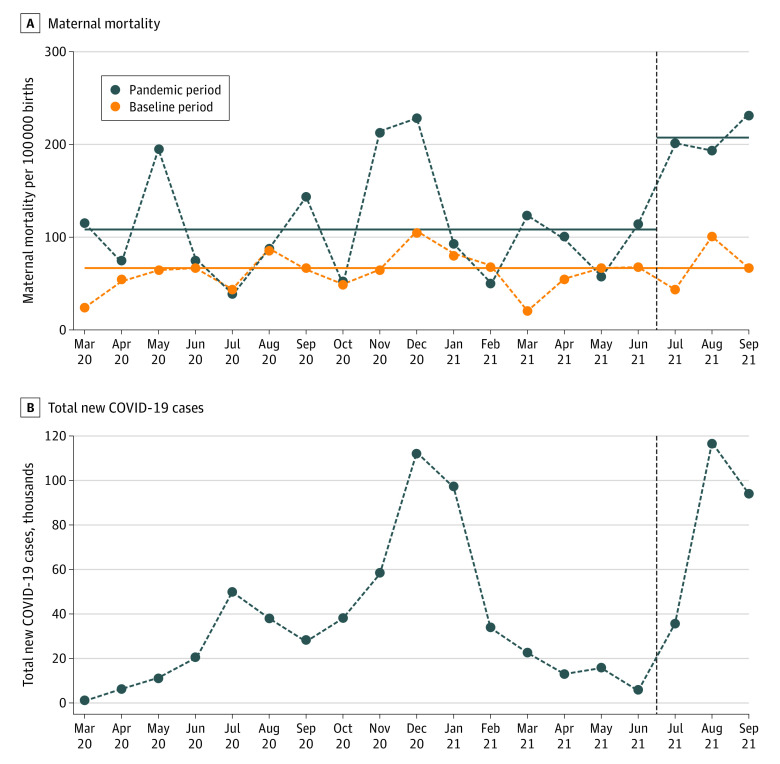
Maternal Mortality by Period and Total New COVID-19 Cases in Alabama Maternal mortality increased during the pandemic periods. The dashed lines are the trends of maternal mortality. The solid lines are the mean maternal mortality by study period. The dashed vertical line denotes the start of the Delta pandemic period.

A decrease in the adequacy of prenatal care was seen in the initial pandemic period compared with the baseline period that was normalized by the Delta pandemic period (Table). An increase in gestational diabetes and pregnancy-induced hypertension was seen during the initial pandemic as well as during the Delta pandemic period (Table). Secular trends for maternal mortality in the baseline period were not significant, therefore, trend-adjusted analysis was not performed.

## Discussion

Findings of this cohort study identified a higher rate of maternal mortality and morbidities during both the initial and Delta pandemic periods compared with the baseline period. The first peak of increase in maternal mortality rate was likely related to mobility restrictions and the subsequent 2 peaks were observed corresponding to the increases in the total COVID cases during that period. The rates of maternal morbidities were higher than those found in a meta-analysis and other shorter duration studies,^4,6^ The association between maternal mortality and morbidity in the pandemic periods and fetal and neonatal outcomes warrants further exploration. Limitations include that this study is based on single-state data, and COVID-19 test results for individual participants were not available in the database.

This study addresses important knowledge gaps, expands on previous findings, and highlights the need for ongoing mitigation and vaccination efforts targeted to pregnant women to help minimize the outcomes of the current pandemic associated with this vulnerable population.

## Notes

Supplement. **eMethods.** Statistical Methods
eReferences
