# Supplementary material for: Trends in Maternal Outcomes During the COVID-19 Pandemic in Alabama From 2016 to 2021
Source: JAMA Netw Open. 2022 Apr 13;5(4):e222681. doi: 10.1001/jamanetworkopen.2022.2681 (PMC9008492; doi:10.1001/jamanetworkopen.2022.2681)
Supplement: Supplement. — eMethods. Statistical Methods eReferences [file jamanetwopen-e222681-s001.pdf]

Supplemental Online Content

Shukla VV, Rahman AKMF, Shen X, et al. Trends in maternal outcomes during the COVID-19 pandemic in Alabama from 2016 to 2021. *JAMA Netw Open.* 2022;5(4):e222681. doi:10.1001/jamanetworkopen.2022.2681

eMethods. Statistical Methods

eReferences

This supplemental material has been provided by the authors to give readers additional information about their work.

© 2022 Shukla VV et al. *JAMA Network Open.*

eMethods. Statistical Methods

The primary outcome of maternal mortality was defined as death of a woman while pregnant or within 42 days of delivery from any cause of the pregnancy or its management, excluding accidental or incidental causes.[1] The adequacy of prenatal care was assessed by Kessner and Kotelchuck indices, encompassing the gestational age at the beginning of prenatal care and the percentage of achieved/expected number of prenatal care visits for gestational age as recommended by the ACOG.[1] The race and ethnicity were reported by the parents/health providers and were identified using the birth/death certificates. The race/ethnicity categories were as per the NCHS classification, and for presentation, the categories were consolidated into 4 groups as Black, Hispanic, White, and Other.[1] The database definitions were used to define other variables and outcomes.[1] CDC daily cases for Alabama[2] were used to identify variations in maternal mortality by frequency of new COVID-19 cases.

© 2022 Shukla VV et al. *JAMA Network Open*.

References

1. Shen X. ALABAMA VITAL STATISTICS 2018. https://www.alabamapublichealth.gov/healthstats/assets/AVS2018.pdf. Published 2018. Accessed 02/16/2021.
2. Trends in Number of COVID-19 Cases and Deaths in the US Reported to CDC, by State/Territory. https://covid.cdc.gov/covid-data-tracker/#trends_dailycases. Accessed 11/21/2021.

© 2022 Shukla VV et al. *JAMA Network Open*.
